# *Zeb1* Is a Potential Regulator of *Six2* in the Proliferation, Apoptosis and Migration of Metanephric Mesenchyme Cells

**DOI:** 10.3390/ijms17081283

**Published:** 2016-08-06

**Authors:** Yuping Gu, Ya Zhao, Yuru Zhou, Yajun Xie, Pan Ju, Yaoshui Long, Jianing Liu, Dongsheng Ni, Fen Cao, Zhongshi Lyu, Zhaomin Mao, Jin Hao, Yiman Li, Qianya Wan, Quist Kanyomse, Yamin Liu, Die Ren, Yating Ning, Xiaofeng Li, Qin Zhou, Bing Li

**Affiliations:** 1 Division of Molecular Nephrology and The Creative Training Center for Undergraduates, The Ministry of Education Key Laboratory of Clinical Diagnostics, School of Laboratory Medicine, Chongqing Medical University, Chongqing 400016, China; littlebottlesky@gmail.com (Y.G.); xianzhaoya@gmail.com (Y.Z.); zhouyuru93@gmail.com (Y.Z.); yjxie@genetics.ac.cn (Y.X.); 18883936591@163.com (P.J.); longyaoshui@gmail.com (Y.L.); liujianingb@gmail.com (J.L.); dongshengni@outlook.com (D.N.); caofen7@gmail.com (F.C.); zhongshilyu@gmail.com (Z.L.); maozhaomin8@gmail.com (Z.M.); lanyxiu@163.com (J.H.); liyimanb@gmail.com (Y.L.); qy.wan@Outlook.com (Q.W.); quistmansa@gmail.com (Q.K.); liuyamin2013@126.com (Y.L.); rendielittle@gmail.com (D.R.); ningyating@outlook.com (Y.N.); happylena18@gmail.com (X.L.); zhouqin@cqmu.edu.cn (Q.Z.); 2 Department of Laboratory Medicine, The First Hospital of Xi’an, Xi’an 710002, China; 3 Undergraduates Class of 2012 Entry, The Fifth Clinical College of Medicine, Chongqing Medical University, Chongqing 400016, China; 4 The First Affiliated Hospital of Chongqing Medical University, Chongqing 400016, China

**Keywords:** *Zeb1*, *Six2*, metanephric mesenchyme cells, cell proliferation, cell apoptosis, cell migration

## Abstract

Nephron progenitor cells surround around the ureteric bud tips (UB) and inductively interact with the UB to originate nephrons, the basic units of renal function. This process is determined by the internal balance between self-renewal and consumption of the nephron progenitor cells, which is depending on the complicated regulation networks. It has been reported that *Zeb1* regulates the proliferation of mesenchymal cells in mouse embryos. However, the role of *Zeb1* in nephrons generation is not clear, especially in metanephric mesenchyme (MM). Here, we detected cell proliferation, apoptosis and migration in MM cells by EdU assay, flow cytometry assay and wound healing assay, respectively. Meanwhile, Western and RT-PCR were used to measure the expression level of *Zeb1* and *Six2* in MM cells and developing kidney. Besides, the dual-luciferase assay was conducted to study the molecular relationship between *Zeb1* and *Six2*. We found that knock-down of *Zeb1* decreased cell proliferation, migration and promoted cell apoptosis in MM cells and *Zeb1* overexpression leaded to the opposite data. Western-blot and RT-PCR results showed that knock-down of *Zeb1* decreased the expression of *Six2* in MM cells and *Zeb1* overexpression contributed to the opposite results. Similarly, *Zeb1* promoted *Six2* promoter reporter activity in luciferase assays. However, double knock-down of *Zeb1* and *Six2* did not enhance the apoptosis of MM cells compared with control cells. Nevertheless, double silence of *Zeb1* and *Six2* repressed cell proliferation. In addition, we also found that *Zeb1* and *Six2* had an identical pattern in distinct developing phases of embryonic kidney. These results indicated that there may exist a complicated regulation network between *Six2* and *Zeb1*. Together, we demonstrate *Zeb1* promotes proliferation and apoptosis and inhibits the migration of MM cells, in association with *Six2*.

## 1. Introduction

The kidney is a vital and complex organ that accomplishes multiple physiological functions in the body, such as metabolic waste excretion, water and electrolyte homeostasis control, acid-base balance and blood pressure maintenance. Nephrons is the major functional units for kidney to perform these tasks [1,2]. During kidney development, nephrons’ formation is mainly decided by the interaction of the ureteric bud (UB) and metanephric mesenchyme (MM) cells [3,4]. It begins at embryonic day 10.5 (E10.5) to E11.0 when the UB starts growing and branching under the induction of MM cells [5], and MM cells aggregate around the branched tips of UB. Then, the MM cells and UB form nephrons by two cellular processes: MET and tubule genesis [6]. These reports indicate that MM cells are the original cells of nephron generation and inductively interact with UB in kidney development [7,8]. In addition, self-renewal (proliferation) and consumption of MM cells determine the formation and complement of nephrons [8,9]. Consequently, the proliferation, apoptosis and migration of MM cells become especially important in the study of kidney development.

*Zeb1*, a transcription factor containing 1117 amino acids, is an EMT marker in cancer metastasis of some tissues including kidney [10,11]. *Zeb1* promotes EMT through suppression of CDH1 (encoding E-cadherin, an epithelial maker) and the microRNA-200 [10]. This process activates transforming growth factor-β1 (TGF-β1) signaling pathway and trigger cancer cell proliferation, invasiveness and stemness out of control [11,12]. In addition, *Zeb1* also plays a critical role in animal organ development [13], cartilage development [14] and regulation of mesenchymal cell proliferation [15]. As an example, loss of *Zeb1* results in MET and reduce the proliferation of progenitor cells at the sites of developmental defects in mouse embryos [15]. However, there is little reference about the concrete role of *Zeb1* in the cellular regulation of MM cells.

*Six2*, a MET-marker, maintains cap mesenchyme multipotent nephron progenitor cells at an undifferentiated state, promotes MM cell proliferation and restrains cell apoptosis during kidney development [8,9,16]. Deficiency of *Six2* depletes cap mesenchyme progenitors, ectopic differentiation, and severe kidney hypoplasia and dysplasia [17,18]. However, EMT and MET are two distinct cellular processes that respectively function in cancer metastasis and development. *Zeb1* and *Six2* are the main markers of these two processes, respectively, but whether there exists a relationship between *Zeb1* and *Six2* in MM cells remains unknown.

Here, we found that *Zeb1* promoted cell proliferation and migration, but suppressed cell apoptosis in MM cells, and *Zeb1* can bind to *Six2* promoter to regulate its transcription by dual-luciferase assay and bioinformatics analysis. Our RT-PCR and Western blot results showed that *Zeb1* increased the expression of *Six2*. Both of *Zeb1* and *Six2* had a high expression level in embryonic kidney at E13.5 and E18.5. These discoveries provided theoretical evidence for further studying the role of *Zeb1*—regulated *Six2* in kidney development.

## 2. Results

### 2.1. Zeb1 Is Highly Conserved and Homologous across Different Mammalians

**Figure 1:**
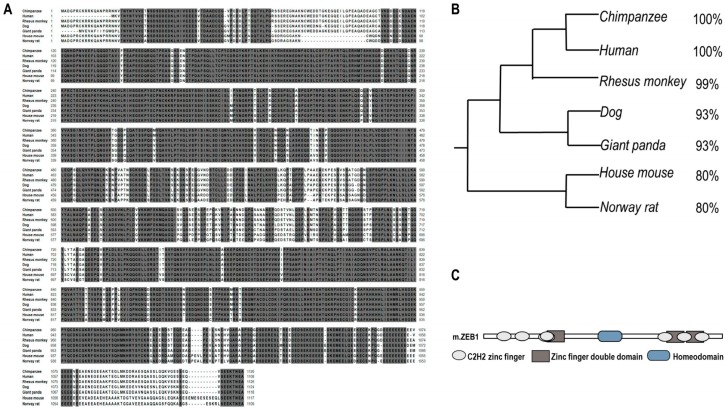
Bioinformatic analysis of *Zeb1* protein. (**A**) Several tracks of entire amino acid sequences of *Zeb1* across different mammal species. NCBI was used to get the sequences that were 1117aa in length and were highly conserved shown in gray shadow representing 100% matched sequences across different species; (**B**) Rooted phylogenetic tree (UPGMA) displayed *Zeb1* is highly homologous among different mammalian. The identity is shown on the right; (**C**) *Zeb1* protein structure contains seven C2H2 zinc finger domains, three zinc finger double domains and one homeodomain.

To analyze the conservative of *Zeb1* protein, we used CLUSTALW online [19]. The *Zeb1* protein is highly conservative and homologous in evolution among mammal species such as Chimpanzee, Human, Rhesus monkey, Dog, Giant panda, Norway rat and House mouse (Figure 1A,B). Additionally, we compared the three types of *Zeb1* function domains (seven C2H2 zinc finger, three Zinc finger double domain and a Homeodomain) in NCBI Protein Database [20]. Then, we found that the structure of *Zeb1* protein across those mammal species is also highly conserved (Figure 1C). 

### 2.2. Zeb1 Promotes the Proliferation and Migration but Inhibits the Apoptosis of MM Cells

As noted above, the function of *Zeb1* in metanephric mesenchymal cells remains unclear during kidney development, so we wonder whether *Zeb1* plays a crucial role in the regulation of these cells. 

**Figure 2:**
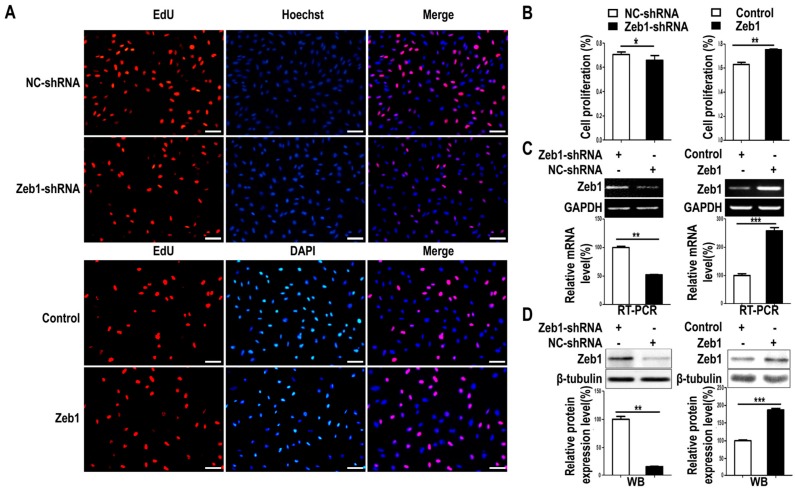
Knock-down of *Zeb1* inhibits mK3 cell proliferation while overexpression of *Zeb1* promotes it. (**A**) Proliferating mK3 cells were labeled with EdU (red) and nucleuses of the whole cells were stained with DAPI or hoechst (blue). The cells were 48 h post-treated with vectors of overexpression or target shRNA or the respective control vectors. The images present were taken by fluorescent microscopy (200×) with a scale bar of 50 μm and the red and blue images were merged to the purple ones; (**B**) EdU positive percentage (EdU %) were quantified. Results were displayed as mean ± SD (*n* = 3). * *p* < 0.05, ** *p* < 0.01, *** *p* < 0.001 negative control vectors; (**C**,**D**) The RT-PCR and western-blot showed the significant efficiency of *Zeb1* overexpression and knock-down. The expression was calculated by scanning gray in Image J software normalized to the internal mRNA control GAPDH or protein control β-tubulin. The result was shown with error bars representing mean ± SD (*n* = 3). * *p* < 0.05, ** *p* < 0.01, *** *p* < 0.001.

**Figure 3:**
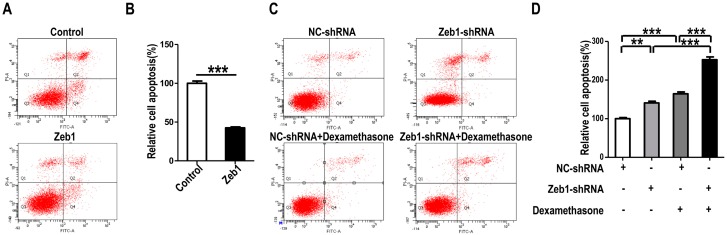
Knock-down of *Zeb1* promotes mK3 cell apoptosis while overexpression of *Zeb1* inhibits it. (**A**) mK3 cell apoptosis was detected by flow cytometry with Annexin V-FITC/PI staining. The number of positive cells double stained by AnnexinV-FITC/PI in mK3 cells transfected with *Zeb1* overexpression vector was dramatically smaller than the control vector. The detection was performed 48 h transfection later; (**C**) mK3 cells were transfected with *Zeb1*-shRNA or negative control shRNA for 20 h. Then one well cells of the two transfected with the same vector was treated with 0.1 μM dexamethasone for 30 h, following by cell apoptosis detection. The number of AnnexinV-FITC/PI-positive cells in *Zeb1* knock-down mK3 cells was significantly larger than the negative control cells. And the dexamethasone at 0.1 μM concentration increased the number of AnnexinV-FITC/PI-positive mK3 cells; (**B**,**D**) Apoptosis rates (Q2% + Q4%) of mK3 cells detected in Figure 3A,B were respectively quantified. Results were displayed as mean ± SD (*n* = 3). ** *p* < 0.01, *** *p* < 0.001 negative control vector.

**Figure A1:**
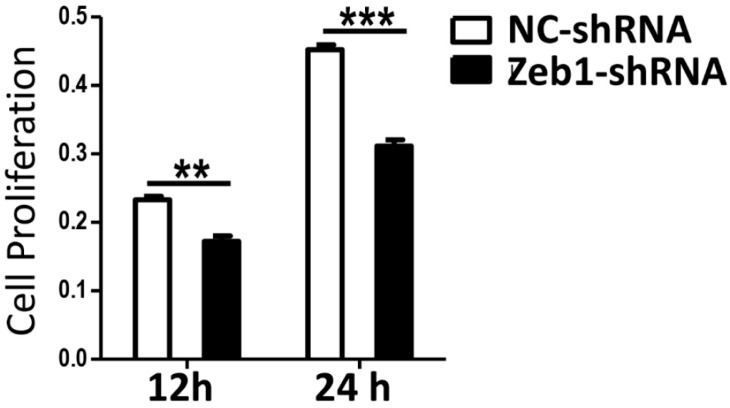
Knock-down of *Zeb1* inhibits mK3 cell proliferation. mK3 cells were 48 h post-treated with vectors of target shRNA or the respective control vector, then the equal 2000 cells were seeded into the 96-well. Proliferating mK3 cells were tested in living cells using MTT at 12 and 24 h. The formazan product was detected by the intensity of absorbance at 590 nm wavelength to indicate cell vitality. The absorbance intensity of *Zeb1* knocked down cells was smaller than the negative control cells at both 12 and 24 h. The absorbance present were examined by a microplate reader and results were displayed as mean ± SD (*n* = 3). ** *p* < 0.01, *** *p* < 0.001 negative control vectors.

To investigate whether *Zeb1* affects the proliferation, apoptosis and migration of MM cells, mK3 cells were used as a cell model. mK3 cells were transfected with *Zeb1* overexpression or knock-down (*Zeb1*-shRNA) vector followed by EdU assay. As illustrated in Figure 2A,B and Figure A1, the ratio of EdU positive cells to the whole cells was promoted in mK3 cells transfected with the overexpression vector compared with the control. However, the ratio was reduced while *Zeb1* was knocked down in mK3 cells. Meanwhile, to find out the effect of *Zeb1* on cell apoptosis of the mK3 cells, we detected the apoptosis of mK3 cells transfected with *Zeb1* overexpression vector, overexpression control vector, *Zeb1*-shRNA or control shRNA (pLKO.1). Then, mK3 cells transfected with *Zeb1*-shRNA or control shRNA (pLKO.1) were treated with 0.1 μM dexamethasone before cell apoptosis analysis. Overexpression of *Zeb1* decreased the rate of mK3 cell apoptosis compared with the control cells (Figure 3A,B). Besides, *Zeb1* silence and dexamethasone treatment increased the apoptosis rate of mK3 cells and knockdown of *Zeb1* increased cell apoptosis induced by dexamethasone compared with the respective control cells (Figure 3C,D). These results demonstrate that *Zeb1* inhibits MM cell apoptosis.

**Figure 4:**
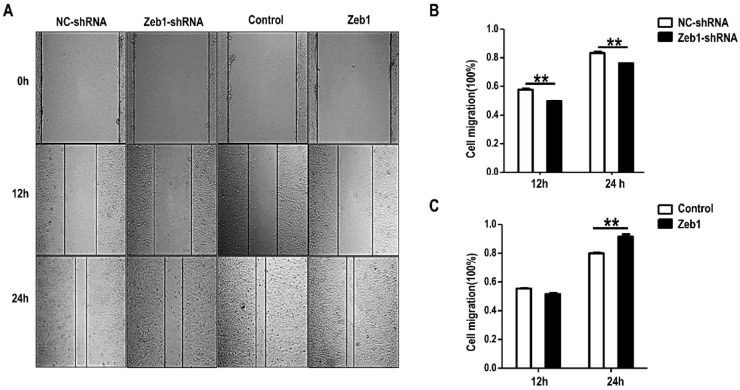
Knock-down of *Zeb1* inhibits mK3 cell migration while overexpression of *Zeb1* promotes cell migration only after 24 h. (**A**) Cell migration of mK3 cells was measured via wound healing assay. The width of wound area was calculated at three time points (0, 12, 24 h) starting from the point when mK3 cells were transfected 48 h later. The transfection way was same with Figure 2. *Zeb1* knock down led to lower apoptosis rate but *Zeb1* overexpression resulted in higher rate, compared with the controls; (**B**,**C**) The quantification of wound healing percentage (100%) was exhibited with the error bars representing mean ± SD (*n* = 3). ** *p* < 0.01, negative control vector.

In addition, we treated mK3 cells with the same methods used in the proliferation assay. Contrarily, we performed Wound Healing Assay and found that deficiency of *Zeb1* resulted in the reduction of wound healing percentage compared with the control (Figure 4A,B). In contrast, mK3 cells transfected with the overexpression vectors shows promotion of cell healing percentage (Figure 4A,C). To some degree, these data concludes that the migration of mK3 cells is advanced by *Zeb1*.

Moreover, to determine the efficiency of *Zeb1* overexpression or knock-down, we conducted the RT PCR and Western-blot. As expected, *Zeb1* was overexpressed and knocked down efficiently (Figure 2C,D). All these findings indicates that *Zeb1* can promotes the proliferation of mK3 cells.

### 2.3. Zeb1 Binds to Six2 Promoter and Up-Regulates Six2 in Metanephric Mesenchymal Cell 

**Figure 5:**
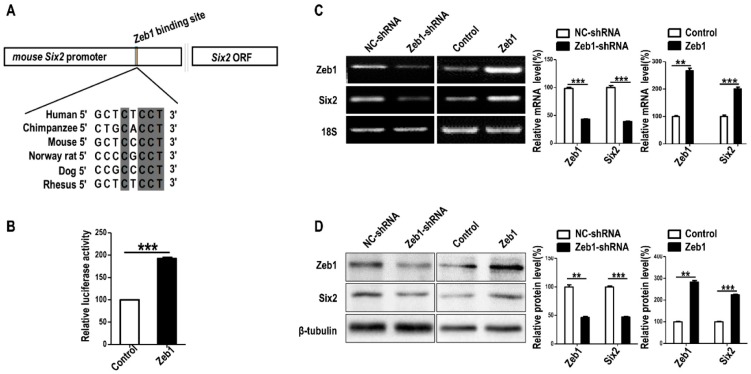
*Zeb1* binds to *Six2* promoter and up-regulates *Six2* in mK3 cells. (**A**) The predicted binding motifs of *Zeb1* to *Six2* promoter, which was acquired from the JASPAR Database. The binding motifs across mammal species are conservatively shown in gray shadow representing the matched sequence among several mammal species, for instance, seven base pairs in eight were matched between mouse and human; (**B**) G401 cells were co-transfected with pGL-SV40 (renilla control), firefly luciferase reporter pcDNA3.1-*Six2* promoter-luciferase, and either the m.*Zeb1* expression plasmid pCDH-copGFP-m.*Zeb1* or the respective control vector. Luciferase activity was assayed using dual luciferase reporter assay 48 h after transfection, normalized to renilla control. The result was analyzed by student’s *t* test and displayed with error bars representing mean ± SD (*n* = 3), ** *p* < 0.01, *** *p* < 0.001; (**C**,**D**) RT-PCR was used to detect the expression of *Six2* at both mRNA and protein level. *Six2* was remarkably decreased in mK3 cells treated with *Zeb1*-shRNA compared with negative control shRNA. Overexpressing *Zeb1* in mK3 cells led to the promotion of *Six2* expression at mRNA level. The expression was quantified by scanning gray in Image J software normalized to the internal mRNA control 18S or protein control β-tubulin. The result was displayed with error bars representing mean ± SD (*n* = 3), ** *p* < 0.01, *** *p* < 0.001.

To find out the mechanism of the cellular regulation in addition to metanephric mesenchymal cells, we performed bioinformatics prediction of the interaction between *Zeb1* and *Six2*. As exhibited in Figure 5A, *Zeb1* binding motifs towards the potential promoter of *Six2* were conserved among mammal species such as Human, Chimpanzee, Mouse, Norway rat, Dog and Rhesus. This result shows it is possible that *Zeb1* can regulate the expression of *Six2 gene* by mediating the transcription of *Six2*. Therefore, we carried out dual-luciferase assay, RT-PCR and Western blot to verify *Zeb1* affected the expression of *Six2*. We found that overexpression of *Zeb1* significantly promotes *Six2* promoter reporter activity (Figure 5B). Additionally, the deficiency of *Zeb1* up-regulated both mRNA and protein expression of *Six2* compared with the control (Figure 5C). In contrast, *Zeb1* overexpression caused the up-regulation of *Six2* at both mRNA and protein level (Figure 5D). It has been reported that *Six2* suppression inhibits cell proliferation, but promotes cell apoptosis in MM cells and up-regulation of *Six2* promotes cell migration [8,17]. These previous studies can explain the phenotype of *Zeb1*.

### 2.4. Zeb1 Regulates Cell Proliferation and Apoptosis of MM Cells by Working with Six2

**Figure 6:**
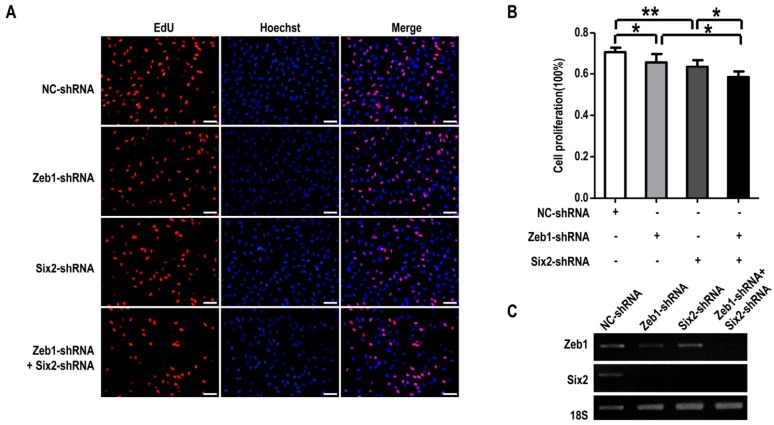
*Zeb1* is required for the proliferation of mK3 cells associated to *Six2*. (**A**) mK3 cells were 48 h post-introduced with control shRNA, *Zeb1*-shRNA, *Six2*-shRNA or both of the two target shRNA. Then EdU assay was performed refering to Figure 2 and the red and blue images were merged to the purple ones; the length of scale bar was 50 μm. Cell proliferation was decreased in mK3 cells treated with either single shRNA or double shRNA of *Zeb1* and *Six2* compared with respective controls; (**B**) The data of proliferation percentage (EdU %) were presented as mean ± SD (*n* = 3). * *p* < 0.05, ** *p* < 0.01, respective controls; (**C**) The significant knock-down efficiency of *Zeb1* and *Six2* was measured by the RT-PCR with the internal control 18S equal.

To clarify the interaction between *Zeb1* and *Six2* in metanephric mesenchymal cells’ regulation, mK3 cells were transfected with negative control shRNA (pLKO.1), *Zeb1*-shRNA, *Six2*-shRNA or both *Zeb1*-shRNA & *Six2*-shRNA followed by EdU assay and apoptosis detection. As shown in Figure 6A,B, mK3 cells introduced with either *Zeb1*-shRNA or *Six2*-shRNA made cell proliferation significantly reduced, compared with the negative control cells. Moreover, mK3 cells transfected with both of *Zeb1*-shRNA and *Six2*-shRNA presented a smaller ratio compared with the mK3 cells transfected with one of the two shRNA. These results declare that down-regulation of *Six2* restrains MM cell proliferation indeed and this suppression was enhanced when *Zeb1* was knocked down with *Six2* down-regulated (Figure 5C).

**Figure 7:**
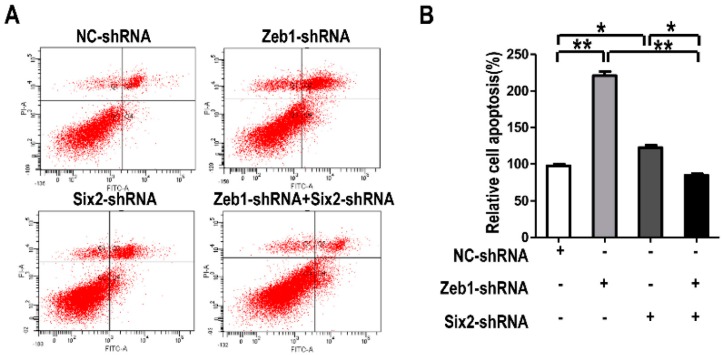
*Zeb1* is required for mK3 cells to survival associated with *Six2*. (**A**) mK3 cells were transfected same with Figure 6 and cell apoptosis was detected by reference to Figure 3. The rate of apoptosis in mK3 cells treated with single target shRNA is higher than the negative control shRNA, while mK3 cells treated double shRNAs had a lower rate than the single targeted mK3 cells; (**B**) Cell apoptosis rate was quantified and presented as mean ± SD (*n* = 3). * *p* < 0.05, ** *p* < 0.01, respective controls.

What is more, we performed cell apoptosis detection in mK3 cells treated the same as in Figure 6. For cell apoptosis, when mK3 cells were introduced with either *Zeb1*-shRNA or *Six2*-shRNA, the rate of cell apoptosis was higher than the control cells. However, it is found that mK3 cells introduced with both of the two shRNAs had a lower apoptosis rate, compared with that transfected by only one shRNA (Figure 7A,B). From these data, it is implied that there is interaction between *Zeb1* and *Six2* in the apoptosis regulation of MM cells. The complicated relationship leads to the different apoptosis trends between single-knock-down of *Zeb1*, *Six2* and double-knock-down of both. 

Meanwhile, to confirm *Zeb1* mediates cell phenotypes above by affecting the expression of *Six2*, we quantified the mRNA expression of *Six2* by RT-PCR. As illustrated in Figure 6C, the knock-down of *Zeb1* or *Six2* was all efficient and *Zeb1* knock-down significantly down-regulated *Six2* expression at the mRNA level.

### 2.5. The Expression Profile of Zeb1 and Six2 in Kidney Development

**Figure 8:**
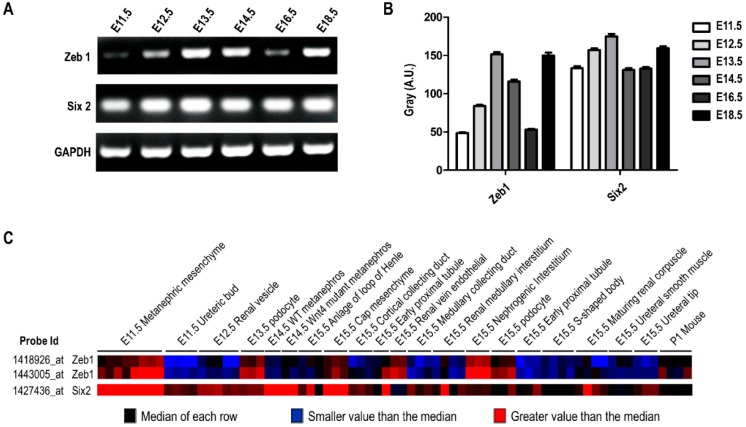
*Zeb1* and *Six2* have a similar expression profile in mouse kidney development. (**A**) RT-PCR was performed using the cDNA library obtained from the embryonic mouse kidney at different embryonic days (E11.5, E12.5, E13.5, E14.5, E16.5, E18.5). The mRNA expression of both genes showed identical variation trends except for E16.5; (**B**) The quantification was analyzed by Gray Scan normalized to the internal control GAPDH and the results were acted as mean ± SD (*n* = 3); (**C**) Bioinformatics analysis of *Zeb1* and *Six2* mRNA expression was performed in GUDMAP Expression Database. It is the microarray data in different cells of developing renal, in which blue represents the median of each row, black means the expression value is smaller than median, while red represents a larger value.

As mentioned above, we found *Zeb1* mediated cell proliferation, apoptosis and migration in MM cells associated with *Six2*, which was fundamental for kidney development. Thus, we wonder whether *Zeb1* or *Six2* was expressed differently in variant stages during kidney development. Accordingly, we conducted RT-PCR using cDNAs of embryonic mouse kidney at different times (E11.5, E12.5, E13.5, E14.5, E16.5, E18.5). As diagramed in Figure 8A,B, the mRNA expression of *Zeb1* and *Six2* changed with the kidney development stage, and the variant tendency of *Zeb1* and *Six2* was identical except for E16.5. This finding suggests that *Zeb1* quite possibly plays an important role during kidney development in relation to *Six2*. Furthermore, we conducted the expression prediction of *Zeb1* and *Six2* by GUDMAP online to analyze *Zeb1* and *Six2* mRNA expression at different times, which presented variant mRNA levels (Figure 8C). 

### 2.6. c-Myc Is Up-Regulated by Zeb1 in MM Cells

**Figure 9:**
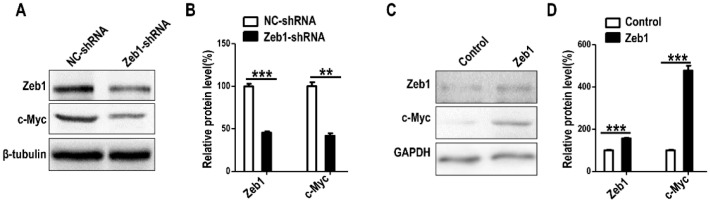
The expression of c-Myc in mK3 cells is regulated by *Zeb1*. (**A**) Western visualization shows that mK3 cells introduced with *Zeb1*-shRNA for 48 h expressed c-Myc less than cells treated with control shRNA at protein level; (**B**) The expression was calculated by scanning gray in Image J software normalized to the internal control GAPDH. The result was displayed with error bars representing mean ± SD (*n* = 3). ** *p* < 0.01, *** *p*<0.001; (**C**) The protein expression of c-Myc was increased in mK3 cells treated with *Zeb1* overexpression for 48 h compared with the control cells (blank vector); (**D**) The expression was quantified the same way as in (**B**).

To verify the mechanism that *Zeb1* mediates MM cells by regulating *Six2*, we checked the protein expression of c-Myc, a transcription factor that has been reported to have interaction with *Six2* during nephrogenesis [21]. The Western-blot data showed that the deficiency of *Zeb1* down-regulated c-Myc (Figure 9A,B) and *Zeb1* overexpression led to the up-regulation of c-Myc (Figure 9C,D). 

## 3. Discussion

*Zeb1*, a transcription factor, is highly conserved and homologous in evolution among different species at the protein level. Here, we first reported the role of *Zeb1* in kidney development and its cellular function in MM cells. Knock-down of *Zeb1* decreased cell proliferation and migration, but increased cell apoptosis in MM cells (Figure 2, Figure 3 and Figure 4 and Figure A1). Deficiency of *Zeb1* down-regulated the expression of *Six2* and c-Myc (Figure 5C,D and Figure 9A,B). Moreover, overexpression of *Zeb1* promotes *Six2* promoter reporter activity in luciferase assay (Figure 5B). On the contrary, overexpression of *Zeb1* leads to the opposite results (Figure 2, Figure 3 and Figure 4, Figure 5C,D and Figure 9C,D). However, double knock down of *Zeb1* and *Six2* decreased cell proliferation more seriously (Figure 6A,B), but did not enhance cell apoptosis in MM cells compared with *Six2* or *Zeb1* knockdown alone (Figure 7A,B). 

Metanephric mesenchyme (MM) cells include the nephron progenitor cells marked by *Six2* and form nephrons by balancing self-renewal and consumption [7,9]. Numerous transcription factors are involved in this process, such as c-Myc and *Six2* [8,17]. In this study, we focused on a transcription factor *Zeb1*, which is widely expressed in development and cancer and acts as an inducer of EMT and a regulator of cell migration in cancer cells [10]. In addition, mutation of *Zeb1* can induce MET and reduce the proliferation of progenitor cells at defects sites of developing mouse embryos [22]. Besides, knock-down of *Zeb1* inhibits cell growth via activating the apoptosis pathway [23], and TXNIP/miR-200/*Zeb1*/E-cadherin signaling pathway is reported to function in beta cell apoptosis [24], which suggests that *Zeb1* plays a crucial role in cell apoptosis. Meanwhile, our results suggested that the resistant of *Zeb1*-depleted mK3 cells to dexamethasone are decreased in comparison to control cells, which implied that *Zeb1* is involved in the apoptosis process that is induced by agent dexamethasone (Figure 3C,D). So, we speculate that *Zeb1* may be a regulator of MET and had an association with MET marker-*Six2* in MM cells. 

Interestingly, knock-down of *Zeb1* decreased cell proliferation and migration but increased cell apoptosis in MM cells as reported in other cells [10,22]. Furthermore, the expression of *Six2* was down-regulated when *Zeb1* was knocked down. Down-regulation of *Six2* represses MM cell proliferation and migration [16,18] but enhances cell apoptosis [8], and overexpression of *Zeb1* leads to the opposite results, reasonably. These suggested that *Six2* is involved in the process that *Zeb1* mediated cell proliferation, migration and apoptosis in MM cells. As is known to us, *Six2* is an essential gene in kidney development and its expression is variant during renal development [25]. We further measured the mRNA expression of *Six2* and *Zeb1* in embryonic mouse kidney in vitro (Figure 8A,B). Although their expression pattern is not completely the same, both of *Six2* and *Zeb1* had high expression at the E11.5 to E13.5 stage, which is a key period in the mouse kidney development process. It has been reported that, in the process of mammalian cell apoptosis, caspases mediate 500–1000 proteins’ cleavage and generate many protein fragments, which can increase the probability of cell apoptosis [26,27,28]. Nevertheless, there are some reports that N-terminal truncated LynΔN that is generated by caspase cleavage has been demonstrated with anti-apoptotic roles [29]. Whatever the concrete role of this fragment in cell apoptosis is, these reports provide a direction for our subsequent study about *Zeb1*, *Six2* and cell apoptosis in embryonic mouse kidney development. Here, our results demonstrated that *Zeb1* depletion decreased *Six2* expression and enhanced cell apoptosis in mk3 cells, but the connection between *Six2* down-regulation and cell apoptosis promotion remains unknown. Because of the limit of time and materials, we did not test the protein expression of *Zeb1* and *Six2* in embryonic mouse kidney in the present study, but we will detect their protein level and further study the connection between *Six2* down-regulation and cell apoptosis promotion during embryonic mouse kidney development. Interestingly, the recent study shows that *Zeb1* can positively regulate the mTOR pathway and maintain its threshold level in wild-type MEFs that is required for Akt-S473 generation [30], and mTOR pathway can rescue many developmental defects of embryos due to the ESCO2 mutant [31], which indicates that the mTOR pathway is essential in development. All these studies indicate that mTOR pathway may be involved in *Zeb1*-regulated renal development.

To find out the regulatory mode between *Zeb1* and *Six2*, we combined the bioinformatics analysis with the dual-luciferase assay results and found that *Zeb1* could bind to *Six2* promoter to enhance the luciferase activity. Therefore, these may prove that *Zeb1* binds to *Six2* promoter to promote *Six2* gene transcription and further promotes gene expression, which is identical to the up-regulation of *Six2* in *Zeb1* overexpressed cells. All these indicated that *Zeb1* might up-regulate *Six2* by transcriptional regulation. Perhaps post-translational modification is another regulation mode, which provides us valuable insight for further research on the regulation mechanism between *Zeb1* and *Six2*.

To make the mechanism clear, we detected the expression of c-Myc, a member of the Myc family of proto-oncogenes expressed in the MM progenitors and which are essential for progenitor cell proliferation and kidney growth [32,33]. Then, we found that a deficiency of *Zeb1* down-regulated c-Myc and overexpression of *Zeb1* up-regulated c-Myc in MM cells (Figure 9A–D). *Zeb1* represses the expression of miR-34a and miR-34b/c, and miR-34a conversely down-regulated *Zeb1* and c-Myc to decrease the migration and invasion of cancer cells [34]. Furthermore, *Six2* mediates the nuclear translocation of Eya1, then Eya1 switches Myc between phosphorylation and dephosphorylation states to regulate MM cell multipotency, proliferation, apoptosis, and so on [21]. Deletion of c-Myc can reduce *Six2*-positive stem/progenitor populations and decrease cell proliferation [33]. Therefore, these evidences demonstrated *Six2* and c-Myc may be new targets of *Zeb1*. However, the role of c-Myc needs to be clarified in further study, and elucidating the regulation network among them appears especially important in kidney development.

**Figure 10:**
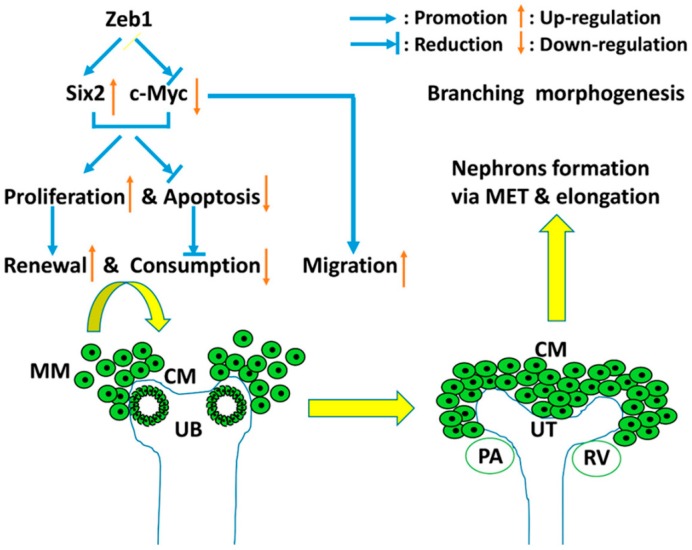
The work model of *Zeb1* in developing embryonic kidney. *Zeb1* promotes MM cell proliferation (cell renewal), cell migration but inhibits cell apoptosis (cell consumption) in association with *Six2* up-regulation and c-Myc down-regulation. As the green cycles show, MM cells migrate and aggregate at UB branches with the induction of UB, then interact with UB. So the MM cell renewal, consumption and migration are essential for the induced interaction between MM cells and UB. These contributes to UB branching morphogenesis and MET and elongation for nephrons formation during embryonic kidney development.

Based on our results and the reported data, we formed a work model to illustrate the regulation between *Zeb1* and *Six2* during embryonic renal development (Figure 10). *Zeb1* promotes MM cell proliferation (cell renewal) and cell migration but inhibits cell apoptosis (cell consumption) in association with *Six2* up-regulation and c-Myc down-regulation. The MM cell renewal, consumption and migration are essential for the induced interaction between MM cells and UB. These contribute to UB branching morphogenesis and MET and elongation for nephrons’ formation in embryonic kidney development. Besides, *Zeb1* and *Six2* have similar expression patterns at different stages of developing kidney (Figure 8A,B). These results further suggested that *Zeb1* is a potential regulator of *Six2* and c-Myc in the proliferation, migration and apoptosis of MM cells.

## 4. Materials and Methods

### 4.1. Bioinformatic Analysis

The species and evolutionary conservation of *Zeb1* proteins was analyzed using Multiple Sequence Alignment by CLUSTALW online [19] and edited in BioEdit software. The amino acid sequences used for analysis were acquired from the NCBI GenPept Database [20]. Moreover, gene expression pattern (Developing Kidney MOE430 Microarray Analysis) of *Zeb1* and *Six2* was obtained from the GUDMAP Expression Database [35]. Additionally, the motifs where *Zeb1* may bind to *Six2* potential promoter (2000bp upstream of transcription start site) was predicted on the JASPAR Database [36], the six promoter sequence (NCBI reference sequence NC_000083.6) was retrieved from the Genbank Database.

### 4.2. Plasmids Construction

The m.*Zeb1* CDS was amplified from the cDNA of C57BL/6 embryonic mouse kidney by PCR using the forward primer: 5′-tagcgtttaaactta GATCATGGCGGATGGCCCCAGG TGTAAGC-3′, the reverse primer: 5′-tggactagtggatcc CTAAGCTTCATTTGTCTTCTCTTCA-3′. The amplification was inserted into the HindIII/BamHI site of pCDNA3.1 (+) (Invitrogen, Carlsbad, CA, USA) using the ligation-independent cloning (LIC) [37,38]. Then the m.*Zeb1* CDS fragment was cut by the NheI/BamHI restriction enzyme and was cloned to the pCDH-CMV-MCS-EF1-copGFP vector (a lentivirus overexpression vector) at the same restriction site using T4 ligation cloning. The m.*Zeb1* shRNA and m.*Six2* shRNA sequences were acquired from the SIGMA ALORICH [39] with m.*Zeb1* target: 5′-CCGGGTCAGTAAACATACCTA-3′; m.*Six2* target: 5′-CCTCCACAAGAATGAAAGCGT-3′. Then anneal the shRNA oligo containing gene target and clone the annealed fragment to pLKO.1 vector at AgeI/EcoRI site according to the protocols [40] pGL3-basic vector (Promega, Madison, WI, USA) and pRL-SV40 was purchased from Promega. The promoter of murine *Six2* was amplified from C57BL/6 mouse genomic DNA by PCR using the forward primer: CGTGCTAGCCCGGGCTATTTCCCAGGTCCCCTGGAATCCT and the reverse primer: CCGGAATGCCAAGCTCTTGCAGCTTTTTTAATAATATTAT. Then the fragment was inserted into the XhoI/HindIII site (upstream of fly luciferase gene) of the pGL3-luciferase vector to create pGL3-*Six2* promoter-luciferase using LIC. All of these recombined vectors were sequenced and aligned in the NCBI Nucleotide Blast Database.

### 4.3. Cell Culture and Transfection

The 293T cell and mK3 cell (a cell line cloned from mouse and representing the un-induced differentiation stage of metanephric mesenchyme [41,42]) were cultured in DMEM medium (Gibico, Carlsbad, CA, USA) with 10% FBS (Gibico, Carlsbad, CA, USA), 1000 units/mL of penicillin and 1000 μg/mL of streptomycin in 37 °C, 100% humidity and 10% CO_2_. The G401 cell were cultured in the same condition except the different base medium ATCC-formulated McCoy’s 5a Medium Modified (Cat. No.30-2007, ATCC). When the mK3 cell grew to 60% confluent in 6-well plates, lentivirus mediated cell transfection was performed, with the assist of polybrene (8 μg/mL). The lentivirus was packed in HEK293T cell line with 10 μg recombined vector in 10 cm dish, then it was harvested to infect mK3 cells after 48 h. The pCDH-CMV-MCS-EF1-copGFP-m.*Zeb1* CDS, pLKO.1-m.*Zeb1* shRNA, pLKO.1-m.*Six2* shRNA and the corresponding control vectors were transfected alone while the m.*Zeb1* shRNA was co-transfected with the m.*Six2* shRNA in another well.

### 4.4. RNA Extraction and RT-PCR

The total RNA was isolated using Trizol reagent (Invitrogen, Carlsbad, CA, USA) from 48 h post-transfected mK3 cells and the kidneys of C57BL/6 embryonic mice at different developmental stage. The cDNA was synthesized using the Invitrogen RT kit according to the manufacturer’s protocol (Invitrogen). The mRNA expression level of *Zeb1*, *Six2* was detected by RT-PCR at 60 °C annealing temperature with *Zeb1* real time PCR sense primer: 5′-CGAGTCAGATGCAGAAAATGAGCAA-3′ and the anti-sense primer: 5′-ACCCAGACTGCGTCACATGTCTT-3′; *Six2* sense primer: 5′-GCCTGCGAGCACCTCCACAAGAAT-3′ and the anti-sense primer: 5′-CACCGACTTGCCACTGCCATTGAG-3′. The expression were normalized to the internal control (18s or GAPDH) and were quantified by Gray Scan using Image J software.

### 4.5. Western Blotting

mK3 cells that were transfected for 48 h in 6-well plates were washed with PBS, pH 7.4 three times. Then, the cells were lysed with 300 μL of 1% SDS lysis buffer. The lysed complex were collected and boiled at 95 °C in a water bath for 10 min followed by centrifuging at 12,000 rpm for 10 min to gather the proteins in supernatant. The concentration of proteins was measured using the Pierce BCA Protein Assay Kit (Thermo Scientific, Waltham, MA, USA) based on the manufacturer instructions. 30 μg of each sample was used to conduct western in reference to the previous studies [8,43]. The primary antibodies respectively against *Six2*, *Zeb1*, c-Myc, β-tubulin were efficient with proper dilution (1:700; 1:800; 1:1000; 1:4000; Proteintech, Chicago, IL, USA).

### 4.6. 5-Ethynyl-2′-deoxyuridine (EdU) Assay

mK3 cells were transfected with *Zeb1* overexpression vectors or *Zeb1*-shRNA vectors or co-transfected with *Zeb1*-shRNA and *Six2*-shRNA vectors by lentivirus. Forty-eight hours later, the treated mK3 cells were seeded onto 96-well plates (10 thousand cells each well) and grew for about 8 h so that the cells were well adherent. Then the proliferation of mK3 cells was detected using the EdU DNA Proliferation in Detection kit (RiboBio, Guangzhou, China) according to the manufacturer instructions.

### 4.7. MTT Assay

The MTT assay was carried out to test the changes of cellular viability. About 2000 mK3 cells transfected with *Zeb1*-shRNA or NC-shRNA were seeded in 96-well plates with five replicates. 12 h and 24 h later, the medium was aspirated, the cells were incubated with 100 μL fresh medium containing 0.5 mg/mL MTT for 4 h. Then, the medium was discarded and 100 μL of dimethyl sulfoxide was added into the well to dissolve the resulting formazan crystals. The absorbance was detected using a microplate reader (MULTISKAN GO, Thermo Scientific, Waltham, MA, USA) at a test wavelength of 590 nm. The average intensity of absorbance in relation to the formazan product indicated the number of cultured living cells with the equal cells at 0 h.

### 4.8. Flow Cytometry Assay and Reagent 

The apoptosis of mK3 cells was determined via the Annexin V-fluorescein isothiocyanate (FITC) Apoptosis Detection Kit (KeyGEN BioTECH, Nanjing, China). mK3 cells were transfected when the cells grew to about 60% confluence and 0.1 μM dexamethasone was selected as an inducing agent in mK3 cells transfected with *Zeb1*-shRNA or negative control shRNA for 20 h [44,45,46]. Then, the cells were harvested 48 h later with EDTA-free trypsin. The cells were numbered by cell counting board and pipetted about 1 million for the later treatment according to the manufacturer instructions. The flow cytometry apoptosis detection was operated by the Institute of Pediatrics in Children’s Hospital of Chongqing Medical University. 

### 4.9. Wound Healing Assay 

mK3 cells were plated in 6-well plates in Dulbecco’s modified Eagle’s medium (DMEM) supplemented with 10% FBS. When cells confluence reached about 60%, pCDH-copGFP-*Zeb1* CDS, pLKO.1-*Zeb1*-shRNA, or blank vector (pCDH-copGFP or pLKO.1) were introduced into mK3 cells. The monolayer cells were generated scratch wounds using a pipette tip when cells grew to 95%–100%, then washed twice with PBS, pH 7.4 to wipe off cell debris. Cells were incubated in completed DMEM in 37 °C, 100% humidity and 5% CO_2_. The images of wound width were taken at different time points (12 and 24 h) using a fluorescence microscope (ECLIPSE Ti-s, Nikon, Tokyo, Japan). Four images were collected from independent selected fields of each sample, and the width of wound areas were calculated by 2014 Microsoft PowerPoint.

### 4.10. Dual-Luciferase Assay

The G401 cell (a human tumor epithelial cell of Caucasian male, aging 3 months) were cultured in 24-well plate (0.1 million each well) for 24 h and then transiently co-transfected with pCDH-m.*Zeb1*, pGL3-*Six2* promoter-luciferase (500 ng/well) and plasmid pRL-SV40 (10 ng/well) utilizing Polyetherimide (PEI) (23966-2, polysciences, Warrington, PA, USA). 48 h later, luciferase activity was assayed using Dual-Luciferase Reporter assay kit (Promega). Levels of firefly luciferase were standardized to those of Renilla.

### 4.11. Embryonic Mouse Kidney Isolation

Embryonic kidneys were isolated from the C57BL/6 mouse embryos at different developmental stage (E11.5, E12.5, E13.5, E14.5, E16.5, E18.5) as described [47]. The process was performed in PBS, pH 7.4 under a microscope and the kidneys were stored at −80 °C or put in Trizol solution for total RNA extraction.

### 4.12. Statistical Analysis

All experiments were performed independently three times and the results were presented as the mean ± standard error of the mean (SEM) or SD. Data were assessed for the statistical significance using student’s *t* test. The GraphPad Prism 5 software (GraphPad, San Diego, CA, USA) was used to evaluate the statistical results. Statistical differences were considered significant with * *p* < 0.05, ** *p* < 0.01, *** *p* < 0.001.

## 5. Conclusions

The results shown in this study indicate that *Zeb1* up-regulates *Six2* and promotes proliferation and apoptosis and inhibits the migration in MM cells.

## Abbreviations

*Zeb1*	Zinc finger E-box-binding homeobox 1
MM	Metanephric mesenchymal
UB	Ureteric bud
PA	Pre-tubular aggregate
UT	Ureteric tree
RV	Renal vesicles
CM	Condensed/cap mesenchyme
TGF-β	Transforming growth factor-β
FITC	Fluorescein isothiocyanate fitc
HEK	Human embryonic kidney
MET	Mesenchymal-epithelial-transition
EMT	Epithelial- mesenchymal-transition
DMEM	Dulbecco’s modified Eagle’s medium
